# Response to imatinib in villonodular pigmented synovitis (PVNS) resistant to nilotinib

**DOI:** 10.1186/2045-3329-3-8

**Published:** 2013-05-13

**Authors:** Silvia Stacchiotti, Flavio Crippa, Antonella Messina, Silvana Pilotti, Alessandro Gronchi, Jean Y Blay, Paolo G Casali

**Affiliations:** 1 Adult Mesenchymal Tumor Medical Oncology Unit, Department of Cancer Medicine, Fondazione IRCCS Istituto Nazionale Tumori Milan, Milan, Italy; 2 Department of Nuclear Medicine, Fondazione IRCCS Istituto Nazionale Tumori Milan, Milan, Italy; 3 Department of Radiology, Fondazione IRCCS Istituto Nazionale Tumori Milan, Milan, Italy; 4 Department of Pathology and Molecular Biology, Fondazione IRCCS Istituto Nazionale Tumori Milan, Milan, Italy; 5 Department of Surgery, Fondazione IRCCS Istituto Nazionale Tumori Milan, Milan, Italy; 6 Department of Medicine, Centre Léon Bérard, Lyon, France

**Keywords:** Pigmented villonodular synovitis, PVNS, Imatinib, Targeted therapy

## Abstract

**Background:**

Pigmented villonodular synovitis (PVNS) is a rare locally aggressive tumor. PVNS is characterized in most cases by a specific t(1;2) translocation, which fuses the colony stimulating factor-1 (*CSF1*) gene to the collagen type VIa3 (COL6A3) promoter thus leading through a paracrine effect to the attraction of non-neoplastic inflammatory cells expressing CSF1-receptor. Imatinib is a tirosin-kinase inhibitors (TKI) active against CSF1-receptor whose activity in naïve PVNS was already described. We report on two PVNS patients who responded to imatinib after failure to nilotinib, another CSF1-receptor inhibitor.

**Methods:**

Since August 2012, 2 patients with progressive, locally advanced PVNS resistant to nilotinib (Patient 1: man, 34 years; Patient 2: woman, 24 years) have been treated with second-line imatinib 400 mg/day. Both patients are evaluable for response.

**Results:**

Both patients are still on treatment (7 and 4 months). Patient 1 had a dimensional response by MRI after 2 months from starting imatinib, together with symptomatic improvement. In Patient 2 a metabolic response was detected by [18F]fluorodeoxyglucose–positron emission tomography (PET) at 6 weeks coupled with tumor shrinkage by MRI, and symptomatic improvement.

**Conclusions:**

Imatinib showed antitumor activity in 2 patients with nilotinib-resistant PVNS. This observation strengthen the idea that targeted agent with similar profile can give a different clinical result, as already described for gastrointestinal stromal tumor (GIST) patients treated with the same agents. Molecular studies are needed to clarify the biologic mechanism(s) underlying the response.

## Introduction

Pigmented villonodular synovitis (PVNS) is a rare locally aggressive tumors which generally arise from the synovium in the knee and foot of young individuals. Standard treatment is surgery, but in a proportion of cases surgery fails to obtain definitive disease control [1]. Even if PVNS is a locally destructive benign process and very few patients die of the disease, the resulting functional impairments can be substantial. The activity of imatinib in PVNS was first reported by Blay et al. in 2008 [2], and confirmed thereafter in a retrospective series of 27 patients, with RECIST partial response (PR) in 19% and stable disease in 74% [3]. Imatinib antitumor effect is thought to be mediated by blockade of the colony stimulating factor-1 receptor (CSF1R). In fact, colony stimulating factor-1 (CSF1), i.e. CSF1R ligand, is overexpressed in PVNS due to a specific t(1;2) translocation, which fuses the *CSF1* gene to the collagen type VI a3 (COL6A3) promoter. CSF1, in turn, attracts non-neoplastic inflammatory cells expressing the CSF1 receptor (CSF1R) through a paracrine effect [4]. CSF1 is an inflammatory mediator which is present together with its receptor (CSF1R) in human synovia. Notably, CSF1 expression was demonstrated at protein and mRNA level both in translocated and in non translocated PVNS [5].

## Case report

**Figure 1:**
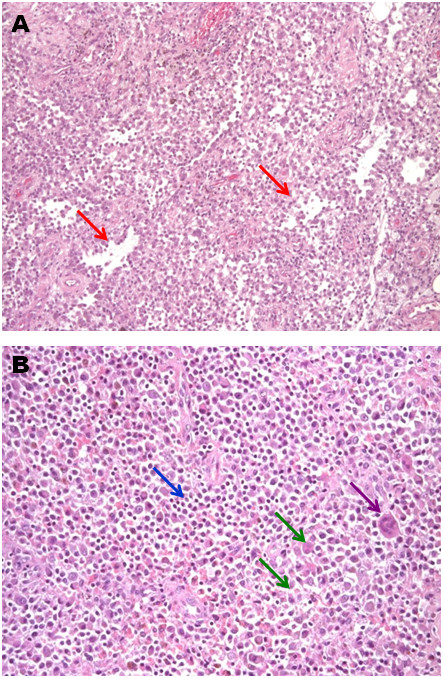
**Path evaluation of Patient 1, Hematoxilin and Heosin-stained (H&H) section.** Tumor biopsy consistent with the diagnosis of PVNS. Histology shows a tumor characterized by a cleft-like space growth lined by synovial-like cells (panel **A**, 50x original magnification, red arrow) and a mixed cellular component made-up of mononuclear round small and large discohesive cells (panel **B**, 100x magnificent magnification, green arrows), rare osteoclast-like giant cells (panel **B**, violet arrow), inflammatory cells (panel **B**, blue arrow).

**Figure 2:**
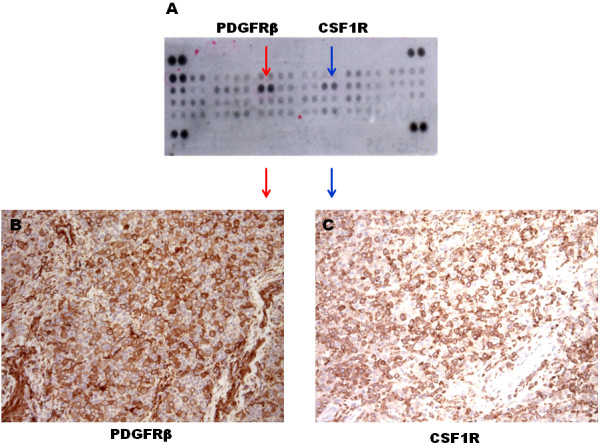
**Path and phospho-receptor tirosine kinase (phRTK) array evaluation of Patient 1.** The phRTK array analysis showed PDGFRB and CSFR1 activation, as indicated in panel **A** by the red and blue arrows, respectively. Immunohistochemistry resulted positive for platelet-derived growth factor receptor beta (PDGFRB) and colony-stimulating-factor1-receptor (CSF1R) expression (panel **B** and **C**, respectively).

The first patient was a 34 years old male, carrying a left knee diffuse-type PVNS treated with debulking surgery elsewhere in 2007 and 2010. As shown in Figure 1, pathologic evaluation of the surgical specimen detected a tumor characterized by a cleft-like space growth lined by synovial-like cells, and a mixed cellular component made-up of mononuclear round small and large discohesive cells, rare osteoclast-like giant cells, inflammatory cells. The mononuclear large cells evaluated by immunohistochemistry (PDGRB: Cell Signaling, clone 4564s; CSF1R: Santa Cruz Biotechnology, clone sc-692) looked positive for CSF1R expression and platelet-derived growth factor receptor beta (PDGFRB) (Figure 2, panel C and B, respectively). The phospho-receptor tirosine kinase (phRTK) array using pair matched cryopreserved material detected PDGFRB and CSFR1 activation. When the disease progressed locally, the patient refused mutilating surgery. Nilotinib was started within a European phase 2 clinical study in July 2011. Tumor size did not change and disease-related symptoms did not improve. The disease progressed after 12 months. In the lack of any alternative standard option, imatinib 400 mg/day was introduced in August 2012. Subjective improvement was seen as from a few weeks of therapy. A dimensional response was confirmed after 2 months by magnetic resonance imaging (MRI). Patient is currently on treatment and progression-free at 7 months.

**Figure 3:**
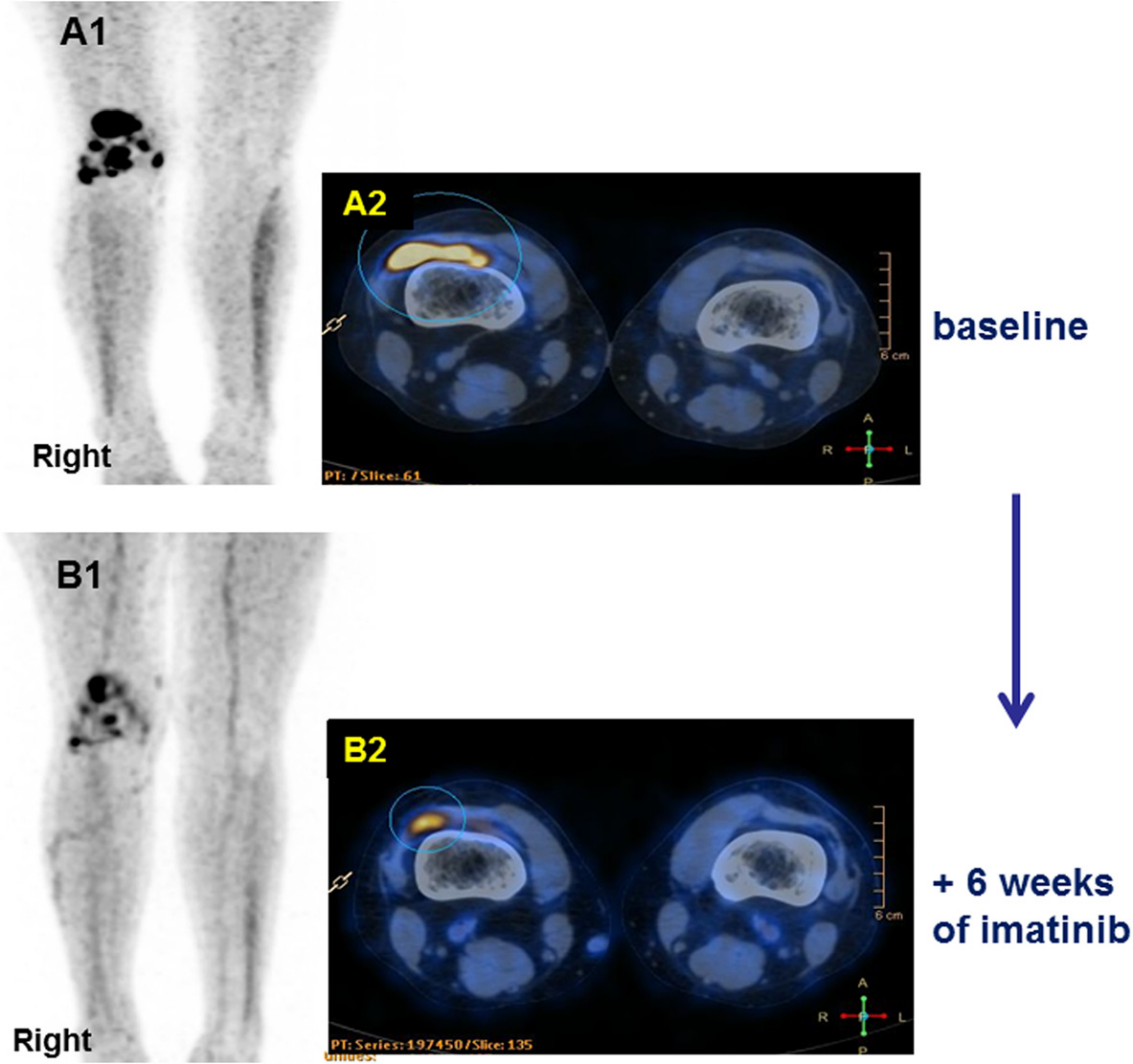
**[18F]fluorodeoxyglucose (FDG) PET/CT tumor assessment of Patient 2.** Baseline PET/CT maximum projection image (MIP) (panel **A1**) showed abnormal FDG focal uptakes in the right knee PVN with a SUVmax of 14.7 g/ml, as detailed by fused PET/CT transaxial slice (panel **A2**). After 6 weeks of treatment, PET/CT MIP (panel **B1**) and fused PET/CT transaxial slice (panel **B2**) showed a marked decrease of tumor FDG uptake (SUVmax 4.1 g/ml, i.e. 72% reduction).

**Figure 4:**
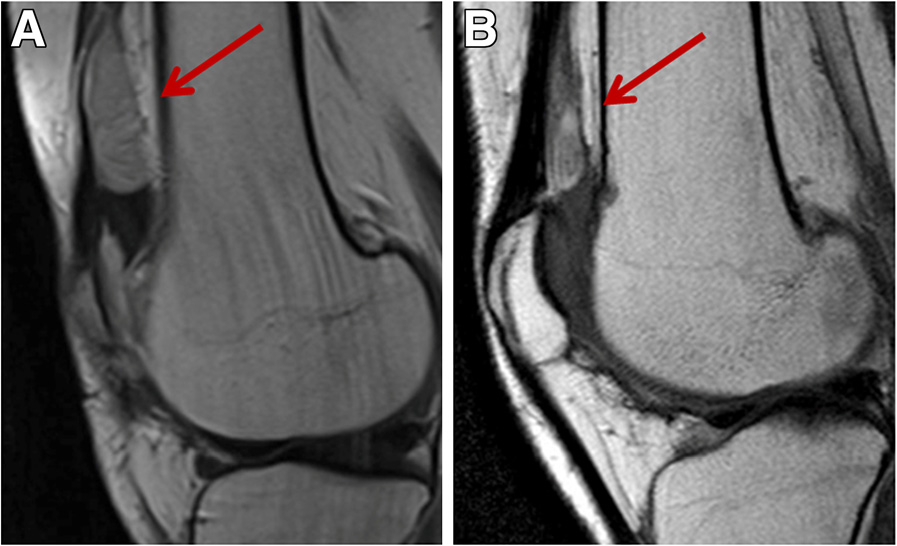
**Magnetic resonance imaging (MRI) tumor assessment of Patient 2.** Sagittal contrast enhanced (ce) TSE T1 weighted (w) MRI images at baseline showed a lesion located behind the rotula (panel **A**). After 2 months of imatinib a decrease in tumor size was detected (panel **B**).

The second patient was a 24 years old female. Her PVNS affected the right knee and was treated with several surgeries from 2002. Due to symptomatic and radiological local progression, she entered a prospective study on nilotinib in February 2012. Slow disease progression was observed, and nilotinib was stopped after 6 months. Imatinib 400 mg/day was started in October 2012. At that time, this patient had functional limitations, pain and local inflammatory signs. After few weeks of imatinib, symptomatic improvement was observed and patient stopped pain therapy. [18F]fluorodeoxyglucose–positron emission tomography (PET)/CT taken after 6 weeks of imatinib confirmed the response. In Figure 3, baseline PET/CT showed a right knee PVNS marked by a maximum standard uptake value (SUV-max) of 14.7 g/ml at the most metabolically active nodule. After treatment, PET/CT (Figure 3) detected an objective response marked by 72% decrease in the tumor metabolic activity (SUV-max 4.1 g/ml). The response was further confirmed by MRI, as described in Figure 4, with evidence of tumor shrinkage and improvement of the synovial effusion. Patient is well after 4 months and treatment is ongoing.

This analysis was approved by the Institutional Ethics Committee.

## Discussion

We report on objective tumor responses we observed in two patients with locally advanced diffuse-type PVNS whom we treated with imatinib following lack of response to nilotinib. In the first case, response to imatinib was observed in a patient that initially got a tumor disease stabilization under nilotinib and further progressed (i.e. secondary resistance to nilotinib), while the second patient never responded to nilotinib (i.e. primary resistance to nilotinib).

Imatinib is a tyrosine-kinase inhibitor with activity against BCR-ABL, KIT, PDGFR, FLT3, RET, and CSFR1. No prospective studies on imatinib in PVNS are available, while a European phase 2 study on nilotinib, another CSF1R inhibitor, was completed in 2012, and results were preliminarily reported with [6]. At a median follow-up of about 10 months, best response was a RECIST PR in one out of 49 patients treated with nilotinib (7%), for a 12-week progression free rate (PFR) of 88.9%. The assumption was that the two agents would have displayed the same pattern of antitumor activity in this rare disease, given their similar molecular profile and their ability to inhibit CSFR1 at similar concentrations [7]. However, the observation of tumor responses to imatinib in nilotinib-resistant PVNS patients is challenging and deserve further investigations.

One hypothesis may be a less favorable pharmakokinetics of nilotinib in these patients, compared to imatinib. Besides, a molecular investigation of these patients would be very helpful to look at polymorphisms of other targets of imatinib and nilotinib in particular KIT and PDGFR. Indirect effects of imatinib on tumor progression, mediated by specific activities on tyrosine kinase receptors other than CSM1R cannot be excluded in these models. In fact, also PDGFRB was found to be activated in the two cases we describe here. This differential activity of imatinib and nilotinib may also be related to differential biochemical activity of these agents on CSFR1. Indeed the side effect profile of nilotinib and imatinib are slightly different, eg with more periorbital oedema with imatinib. This indicates that imatinib has a differential activity on some targets, including possibly CSFR1. Finally, the immune response hypothesis should be also evaluated. In fact, CSFR1 is an inflammatory mediator and can work also as quite a potent blocker of the immune response through the dendritic cells [8], and the NK cells [9]. In addition to selectively inhibit the tumor cells, imatinib was found to be able to induce indirect effects on immune system by stimulating autoimmune response. Therefore, it is tempting to speculate that the action of imatinib in PVNS could be also related to this indirect effect, already reported for gastrointestinal stromal tumor (GIST) patients [10,11]. In diffuse-type PVNS this effect could be even more relevant given the significant amount of inflammatory component detectable in this disease.

## Conclusion

A tumor response to imatinib was seen in two patients with progressive nilotinib-resistant PVNS.

This observation strengthen the notion that targeted agents with an expected similar molecular profile can show a different clinical activity, as already described for GIST patients treated with the same agents. Molecular studies are needed to better understand the molecular basis for the activity of imatinib in this disease.

## Consent

Written informed consent was obtained from the patients for publication of this Case Report and any accompanying legend. A copy of the written informed consents are available for review by the Editor-in-Chief of this journal

## Competing interest

Stacchiotti S – Novartis Farma: research funding; travel coverage. Crippa F – Novartis Farma: married to a full time officer employee. Messina A, Pilotti S: the authors have declared no conflicts of interest. Gronchi A, Blay JY, Casali PG – Novartis Farma: honoraria, advisory, research funding; travel coverage.

## Authors’ contributions

SS, SP and AG contributed case material, and contributed to the conception and design, to the analysis and interpretation of data, to manuscript drafting. FC and AM carried out the radiological evaluation and contributed to the analysis and interpretation of data, to manuscript drafting. JYB and PGC contributed to the analysis and interpretation of data, to manuscript drafting. All the authors read and approved the final manuscript.

Paper was supported by grants from Associazione Italiana per la ricerca sul Cancro (AIRC): IG 10300.
